# UMI-nea: a fast, robust tool for reference-free UMI deduplication and accurate quantification

**DOI:** 10.1093/bioinformatics/btaf514

**Published:** 2025-09-17

**Authors:** Jixin Deng, Jingxiao Zhang, Song Tian, John DiCarlo, Hong Xu, Samuel J Rulli, Jonathan M Shaffer, Vikas Gupta, Toeresin Karakoyun

**Affiliations:** Research and Development, QIAGEN Sciences Inc., Frederick, MD, 21703, United States; Research and Development, QIAGEN Sciences Inc., Frederick, MD, 21703, United States; Research and Development, QIAGEN Sciences Inc., Frederick, MD, 21703, United States; Research and Development, QIAGEN Sciences Inc., Frederick, MD, 21703, United States; Research and Development, QIAGEN Sciences Inc., Frederick, MD, 21703, United States; Product Management Genomics, QIAGEN Sciences Inc., Frederick, MD, 21703, United States; Research and Development, QIAGEN Sciences Inc., Frederick, MD, 21703, United States; Research and Development, QIAGEN Sciences Inc., Frederick, MD, 21703, United States; Research and Development, QIAGEN Sciences Inc., Frederick, MD, 21703, United States

## Abstract

**Motivation:**

One of the key applications of Unique Molecular Identifiers (UMIs) in high-throughput sequencing is to correct for PCR amplification bias and removal of PCR duplicates, thereby improving quantification in DNA-seq and RNA-seq applications. Accurately grouping error-bearing UMIs that originate from the same input molecule through a UMI deduplication method is a critical step in this process. However, many existing UMI deduplication tools rely on simple Hamming distance comparisons or suboptimal clustering algorithms, often resulting in erroneous UMI groupings, particularly in error-prone long-read sequencing or ultra-high-depth short-read sequencing.

**Results:**

We introduce UMI-nea, a tool that utilizes Levenshtein distance comparisons and a novel clustering approach to optimize multithreading workflows. Compared against three other indel-aware UMI deduplication tools, UMI-nea achieves more accurate UMI groupings with efficient run time. It demonstrates robust performance across diverse sequencing platforms, depths, and UMI lengths. Additionally, UMI-nea incorporates a data-guided adaptive UMI filter, further enhancing quantification accuracy.

**Availability and implementation:**

UMI-nea is available on github https://github.com/Qiaseq-research/UMI-nea.git or Zenodo https://doi.org/10.5281/zenodo.16745758. Sequencing data are stored at https://qiagenpublic.blob.core.windows.net/umi-nea-datasets/.

## 1 Introduction

Unique Molecular Identifiers (UMIs) are crucial in next-generation sequencing (NGS) applications, such as low-allele frequency variant detection, T-cell receptor (TCR) repertoire profiling, and single-cell gene expression analysis. Each UMI is typically a short, random sequence that tags a unique input molecule, which is amplified and sequenced. Distinct UMIs can then be used to estimate different populations of DNA or RNA molecules (Kivioja *et al.* 2011).

A major challenge in UMI-based analyses is the introduction of base errors during library preparation or sequencing, which can alter UMI sequences and impact the accuracy of UMI based estimation. To mitigate this, UMI deduplication is used by grouping reads with similar UMI sequences, assuming that sequences within a small edit distance originated from the same molecule. However, UMI errors increase with higher read depth, longer UMI length, or larger platform-specific error rates, complicating the deduplication process. When reads can be aligned to a reference and alignments are scattered, UMI error correction is limited to local areas. However, for low-complexity libraries or applications lacking a suitable reference, UMI deduplication must handle a significantly larger number of UMI errors.

Most existing UMI deduplication tools use Hamming distance for sequence comparison, which only accounts for substitution errors. While effective for short-read sequencing at moderate depths, this approach is insufficient for applications involving ultra-deep sequencing, where the number of indel errors is non-negligible, or long-read sequencing, which tends to have a higher frequency of indels. It also falls short when longer UMIs are necessary to mitigate UMI collisions (Zurek *et al.* 2020, Andersson *et al.* 2024) or applying structured and varied length UMI designs to reduce non-specific PCR (Micallef *et al.* 2025). While Hamming distance calculations are computationally efficient, with a time complexity of O(1) (Liu 2019), Levenshtein distance computations are significantly more complex, with a worst-case time complexity of O(|a|·|b|) for UMIs of lengths a and b, respectively, posing substantial challenges for implementation.

Beyond sequence comparison, UMI deduplication is also affected by the choice of clustering algorithm (Smith *et al.* 2017, Orabi *et al.* 2019, Zurek PJ *et al.* 2020). Poorly chosen methods can lead to over- or under-clustering, skewing molecule counts. For example, clustering by connected components may merge distinct UMIs, underestimating input molecules. In contrast, insufficient clustering can leave related UMIs ungrouped, leading to excessive singleton reads and inflate counts. Singletons are difficult to interpret (Wang *et al.* 2019), and their inclusion in analyses is often questionable (Peng and Dorman 2023).

To reduce the impact of spurious UMIs, arbitrary thresholds are often applied to remove UMIs with low read counts, but this can lead to data loss in cases of shallow sequencing. A dynamic, data-driven method for threshold selection offers a more flexible solution.

To address these issues, we developed UMI-nea, a robust UMI-only and reference-free deduplication tool designed to enhance error correction efficiency. UMI-nea uses Levenshtein distance for both substitution and indel error correction and uses a novel clustering strategy that supports multithreaded processing, which is difficult for network-based clustering algorithms such as UMI-tools (Smith *et al.* 2017) to achieve. It first identifies founder UMIs, then clusters each UMI to its corresponding founder. This is followed by a statistically guided approach to determine an optimal threshold for filtering erroneous UMIs, thereby improving the quantification accuracy. Together, these features provide an efficient and accurate solution for UMI deduplication, reliably addressing substitution and indel errors and large numbers of erroneous UMIs.

## 2 Methods

*UMI assignment to founder UMIs:* UMI-nea assigns each observed UMI sequence to a founder UMI, assuming UMIs with higher read support are more likely to be founders. The process begins by sorting UMIs by read count, initializing the UMI with the highest read count as a founder. Remaining UMIs are compared to existing founders and classified as either a new founder or a progeny UMI based on Levenshtein distance (Martin and Šikić 2017) with a dynamically determined distance threshold based on UMI length and error rate. The upper limit of the binomial confidence intervals is used to calculate this threshold. A UMI is added to the founder list only if its distance from all existing founders exceeds the threshold; otherwise, it is classified as a progeny.

**Figure 1.**
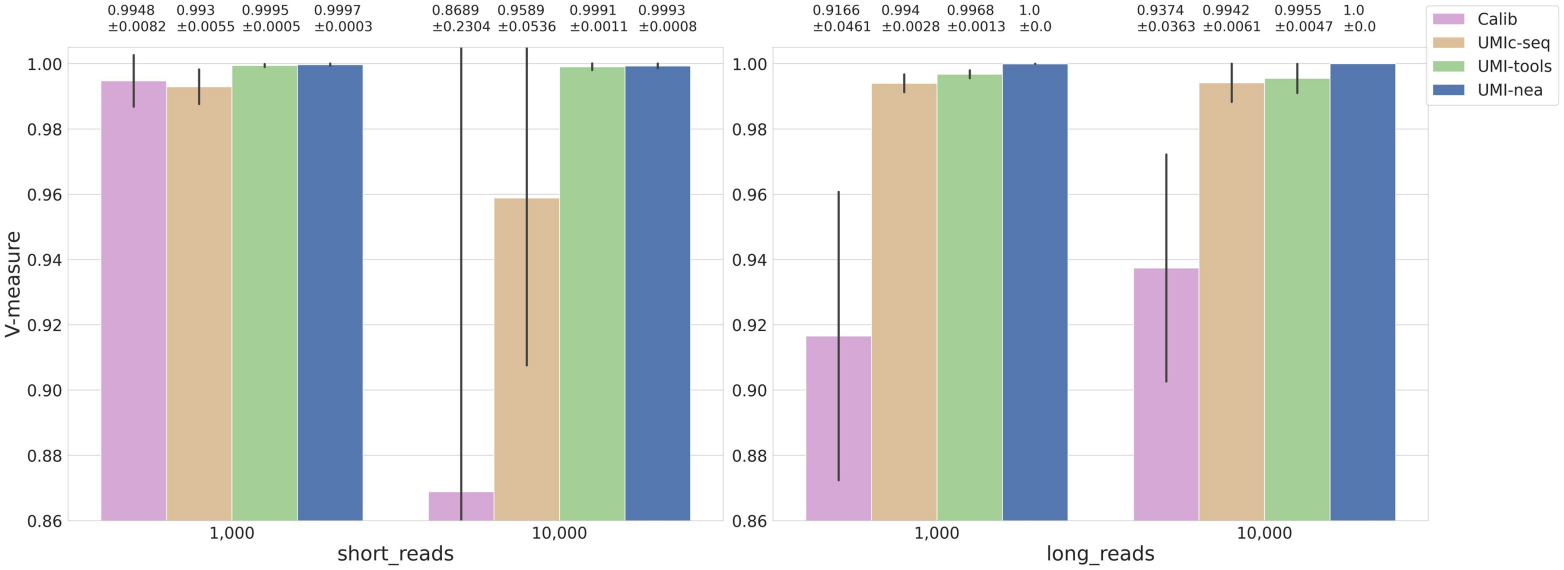
V-measure of UMI clustering performance across four tools on simulated short- and long-read data with 1000 and 10 000 founders.

*Multithreading strategy:* To improve computation efficiency, UMI-nea uses a producer-consumer multithreading model. The producer thread constructs and updates the founder list from the first batch of UMIs, while multiple consumer threads concurrently compare UMIs from subsequent batches to founders and store candidate founders, accessing the shared founder list in a thread-safe manner (Fig. 1, available as supplementary data at *Bioinformatics* online). To ensure reproducibility, the founder of a progeny UMI is identified only by the producer thread, after all founders preceding the progeny UMI have been determined. The closest founder UMI is assigned; in cases of ties, the founder appearing earlier in the sorted list prevails. Finally, UMI-nea outputs all founder–progeny clusters.

*Adaptive threshold determination:* UMI-nea uses an adaptive thresholding approach to filter low-confidence UMIs based on their read support. It applies two complementary methods: one detects a “knee” point in the barcode rank plot, and the other fits a negative binomial distribution to the read count per UMI. The selected method depends on the presence and clarity of the knee point. Full methodological details are provided in the Supplementary Methods, available as supplementary data at *Bioinformatics* online.

*Simulation and benchmarking UMI deduplication:* To evaluate clustering accuracy across both short- and long-read platforms, we simulated UMI sequences with substitution and indel errors under two distinct error models, UMI lengths, and founder counts. Illumina-like short-read simulations used 12 and 18 bp UMIs at error rates of 0.001 and 0.005 with an insertion: deletion: substitution ratio of 1:1:40 (Laehnemann *et al.* 2016). Long-read profiles mimicking PacBio/Oxford Nanopore used 25 and 50 bp UMIs at 0.01 and 0.03 error rates with a 1:1:1 ratio. Founder UMI counts were set to 1000 to model single-cell sequencing and 10 000 to simulate low-allele-frequency variant detection scenarios. Progeny read numbers were sampled from a negative binomial distribution (mean = 100), and each parameter set was simulated in triplicate.

UMI-nea was benchmarked against three other indel-aware UMI deduplication tools: Calib, UMIc-seq, and UMI-tools (see Supplementary Methods, available as supplementary data at *Bioinformatics* online), using the V-measure (Rosenberg and Hirschberg 2007) to assess clustering accuracy and by comparing recovered versus true founder counts to identify over- or under-clustering. We also evaluated the accuracy of UMI-nea’s molecule count estimates following adaptive thresholding. A simulation script is provided to reproduce these benchmarks or test custom parameters.

*Evaluation with real TCR sequencing data:* RNA from three cell lines containing seven known TCR clonotypes was spiked into PBMC RNA at four ratios (0.01%, 0.1%, 1%, and 10%), with two replicates per ratio. Libraries were prepared using the QIAseq Targeted RNA-seq Panel for T-cell Receptor (QIAGEN, Frederick, MD) and sequenced on MiSeq and NextSeq 2000 platforms at varying depths. MiXCR (Bolotin *et al.* 2015) was run using its native pipeline. UMI-tools, UMIc-seq, and Calib shared a standardized workflow for UMI extraction, consensus read generation, and TCR clonotype annotation via IMSEQ (Kuchenbecker *et al.* 2015), differing only in the UMI deduplication step (see Supplementary Methods, available as supplementary data at *Bioinformatics* online).

*Benchmarking multithreaded run time:* We evaluated the run time performance of UMI-nea, UMIc-seq, and Calib, three tools that support multithreading, across four thread counts (12, 24, 36, and 48). All benchmarks were executed independently on a Microsoft Azure Standard_D96as_v4 instance (96 vCPUs, 384 GiB RAM). Each tool was configured to use the specified number of CPU threads, and wall-clock run times were recorded for comparative analysis.

## 3 Results

### 3.1 UMI deduplication performance comparison

For both short- and long-read simulated datasets, we report the mean and standard error of the V-measure (Fig. 1) and the percentage of deduplicated UMI clusters relative to the ground truth (Table 1) at two founder count levels. In the short-read setting, UMI-nea and UMI-tools yield comparable V-measures, both outperforming Calib and UMIc-seq. However, UMI-tools overestimates founder counts by 11.1% and 10.9%, indicating under-clustering, compared to 4.5% and 5.8% for UMI-nea. This stronger under-clustering in UMI-tools is further supported by its lower completeness scores (Table 1, available as supplementary data at *Bioinformatics* online). Calib and UMIc-seq show under-clustering at 1k founders but shift to over-clustering at 10k, as reflected by reduced homogeneity scores. Over-clustering may lead to the erroneous merging of UMIs from distinct founders, complicating downstream error correction.

**Table 1.** Percentage of deduplicated UMI clusters relative to ground truth across four tools on simulated short- and long-read datasets with 1000 and 10 000 founders.

	Short reads		Long reads	
Tool/founder	1000	10 000	1000	10 000
calib	113.9%±12.8%	91.0%±34.4	1793.0%±1264.3%	1735.3%±1240.1%
UMIc-seq	104.4%±9.6%	93.5%±39.2%	100.3%±0.6%	101.0%±1.9%
UMI-tools	111.1%±8.5%	110.9%±7.9%	144.4%±12.9%	143.5%±12.5%
UMI-nea	104.5%±6.6%	105.8%±8.6%	100.6%±0.5%	100.7%±0.4%
UMI-nea + threshold^a^	99.93%±0.11%	99.44%±0.58%	99.98%±0.04%	99.98%±0.02%

a After applying adaptive thresholding, UMI-nea further enhances both the accuracy and precision of founder count estimates compared to results based solely on deduplication, as shown in the row above.

In long reads, UMI-nea, UMI-tools, and UMIc-seq all outperform Calib in V-measure, with UMI-nea slightly leading. UMI-nea and UMIc-seq closely match the true founder numbers, deviating by only 0.6%–0.7% and 0.3%–1.0%, respectively, whereas UMI-tools under-clusters by ∼44%, and Calib significantly under-clusters.

While UMI-nea achieves the highest V-measure and produces UMI cluster counts closest to the true founder numbers in most datasets, it also exhibits the lowest variability in both metrics. In contrast, other tools show greater fluctuation. A key feature of UMI-nea is its adaptive thresholding mechanism, which filters out low-support UMI groups likely arising from uncorrected errors. This step significantly improves quantification accuracy, reducing absolute deviations from 4.5%, 5.8%, 0.6%, and 0.7% to 0.07%, 0.56%, 0.02%, and 0.02%, and standard errors from 6.6%, 8.6%, 0.5%, and 0.4% to 0.11%, 0.58%, 0.04%, and 0.02%, respectively, representing more than a 10-fold gain in both accuracy and precision.

### 3.2 Evaluation of real TCR sequencing data

We evaluated four UMI deduplication tools alongside MiXCR using RNA-seq libraries generated from cell lines with known T-cell receptor (TCR) sub-gene clonotypes. To assess quantification accuracy, we applied two criteria: (i) the estimated molecule counts across four spike-in libraries should reflect the expected 10-fold difference based on known input ratios, and (ii) the counts should be consistent across technical replicates sequenced on two different platforms (MiSeq and NextSeq 2000), both of which provided sufficient sequencing depth.

All four UMI-deduplication tools, along with MiXCR, successfully recovered the expected 10-fold dynamic range (Fig. 2, available as supplementary data at *Bioinformatics* online), although UMIc-seq slightly distorted this range under certain conditions. UMI-nea and MiXCR demonstrated the highest consistency across replicates—both within duplicate libraries and between matched libraries sequenced on MiSeq and NextSeq—exhibiting the lowest coefficients of variation, calculated as the ratio of the standard deviation to the mean of molecule estimates for each of the seven clonotypes within each replicate set. This was followed by UMI-tools and Calib, while UMIc-seq showed markedly higher variability (Fig. 3, available as supplementary data at *Bioinformatics* online). Hierarchical clustering analysis further revealed that UMI-nea and MiXCR produced more similar molecule estimates of clonotypes, clustering closely together. In contrast, Calib and UMI-tools formed a second cluster, with UMIc-seq forming a distinct third cluster (Table 4, available as supplementary data at *Bioinformatics* online, Fig. 4, available as supplementary data at *Bioinformatics* online).

### 3.3 Benchmarking multithreaded run time

UMI-nea was benchmarked against Calib and UMIc-seq (Table 2, available as supplementary data at *Bioinformatics* online); UMI-tools was excluded due to its single-threaded design, with run time reported separately (Table 1, available as supplementary data at *Bioinformatics* online). Calib was consistently the fastest across all thread counts, likely due to its alignment-free clustering strategy. UMI-nea was moderately slower but remained comparable in execution time. UMIc-seq was substantially slower, largely due to its alignment-intensive approach. While Calib and UMIc-seq showed limited performance gains with additional threads, UMI-nea’s run time decreased more noticeably with increased parallelization, suggesting better scalability under multithreaded conditions.

## 4 Discussion

UMI deduplication enables accurate quantification of input molecules, provided two key challenges addressed: UMI sequence errors and UMI collisions. UMI-nea is a UMI-only deduplication tool specifically designed to correct both substitution and indel errors within UMIs and demonstrates robust run time efficiency and consistent performance across a wide range of experimental conditions. By not relying on gene or transcript context, UMI-nea offers advantages in algorithmic simplicity and broader applicability, particularly in assays where reference alignment is limited or potentially biased.

However, like all other UMI-only deduplication methods, UMI-nea cannot fully resolve UMI collisions. This is evident in simulations with many founder molecules paired with shorter UMIs, where it shows reduced V-measure and less accurate clustering (Table 3, available as supplementary data at *Bioinformatics* online). This issue is best addressed by experimental design improvements, such as using longer UMIs (Zurek *et al.* 2020, Andersson *et al.* 2024) to increase UMI uniqueness.

Another current limitation is that UMI-nea has not yet been validated on real sequencing data from long-read platforms. Nonetheless, simulation studies indicate that UMI-nea performs particularly well in high-error-rate scenarios, especially those with elevated indel frequencies and longer UMIs, providing strong initial validation of its performance. We plan to apply UMI-nea in future long-read experiments.

## Supplementary Material

btaf514_Supplementary_Data
