# Supplementary material for: UMI-nea: a fast, robust tool for reference-free UMI deduplication and accurate quantification
Source: Bioinformatics. 2025 Sep 17;41(9):btaf514. doi: 10.1093/bioinformatics/btaf514 (PMC12453673; doi:10.1093/bioinformatics/btaf514)
Supplement: btaf514_Supplementary_Data [file btaf514_supplementary_data.zip › UMI-nea_supplemental_Materials.docx]

**UMI-nea: A Fast, Robust Tool for Reference-Free UMI Deduplication and Accurate Quantification**

Jixin Deng^1,*^, Jingxiao Zhang^1^, Song Tian^1^, John DiCarlo^1^, Hong Xu^1^, Samuel J. Rulli^2^, Jonathan M. Shaffer^1^, Vikas Gupta^1^, Toeresin Karakoyun^1^

^1^Research and Development, QIAGEN Sciences Inc., Frederick, MD, USA

^2^Product Management Genomics, QIAGEN Sciences Inc., Frederick, MD, USA

*To whom correspondence should be addressed.

**Table of content**

**Supplementary Methods**

UMI Thresholding Strategy in UMI-nea

Simulation Methods

Tool Parameters for Simulated Data

V-measure Calculation for Simulated Data

UMI Extraction and Clustering for Real TCR Sequencing Data

Post-Clustering Processing for Real TCR Sequencing Data

MiXCR analyses on Real TCR Sequencing Data

**Supplementary** **Table 1** Benchmark Results for Simulation Data (separate file: Supplemental_Table_1.xlsx).

**Supplementary** **Table 2** Run Time Comparison with Multi-threading for Calib, UMI-nea, and UMIc-seq on Simulated Short-read and Long-read data.

**Supplementary** **Table 3** V-measure and the Number of Resulting clusters of UMI-nea Across Different UMI Lengths on Simulated Short-read and Long-read Data.

**Supplementary** **Table 4** Estimated Molecule Counts and Coefficients of Variation for Seven TCR clonotypes Across Different tools (separate file: Supplemental_Table_4.xlsx).

**Supplementary Figure 1** UMI-nea Workflow.

**Supplementary Figure 2** Quantification of 7 clonotypes in Real TCR Sequencing Data with UMI-nea, UMI-tools, UMIc-seq, Calib and MiXCR on Nextseq2000 and Miseq Platform.

**Supplmentary Figure 3** Coefficient of variation (CV) of Estimated TCR Molecule Counts Across Four Technical Replicates for Different Tools.

**Supplementary Figure 4**  Hierarchical clustering and Pearson correlation heatmap of TCR Molecule Count Estimates Across Different Tools.

**Supplementary References**

**Supplementary Methods**

**UMI Thresholding Strategy in UMI-nea**

UMI-nea employs two complementary strategies to determine the minimum read count required for a UMI to be considered a true molecular signal rather than background noise.

The primary thresholding approach in UMI-nea is based on a knee plot. UMI-nea constructs a barcode rank plot by ordering UMIs in descending order of read count (x-axis) and plotting the cumulative read count (y-axis), with both axes normalized to a 0–1 scale to ensure consistency across datasets. Following the method of Zheng et al. (2017), UMI-nea identifies the “knee”, the inflection point that distinguishes high-confidence UMIs from low-confidence ones, using a geometric approach: it calculates the angle formed between the first, last, and each intermediate point along the curve. The point with the smallest angle is selected as the knee, and its corresponding read count is used as the threshold.

In theory, the minimum angle can range from 90°, which occurs when a small number of true UMIs account for the vast majority of reads (with the rest UMIs being low-support noise), to 180°, which indicates a flat curve with uniform read support and no discernible knee point.

If no distinct knee is detected, defined as all computed angles exceeding a conservative threshold (e.g., 120°), UMI-nea switches to a **model-based fallback strategy**. This method fits a negative binomial distribution X∼NB(r,p) to the UMI read count data, with parameters estimated using the method of moments as an approximation to the maximum likelihood estimates:

$\hat{p}=\frac{\bar{x}}{s^{2}}$ and $\hat{r}=\frac{\bar{x}^{2}}{s^{2}-\bar{x}}$

UMI-nea then applies a lower-tail quantile cutoff to exclude low-support UMIs likely arising from sequencing or amplification errors.

By default, UMI-nea prioritizes the knee plot method and resorts to the negative binomial model only when a clear inflection point cannot be identified. Based on our observations, the knee plot is most effective for low-input samples or samples with high reads per UMI, where a distinct inflection point is often present. In these cases, using the knee plot avoids the risk of poor model fitting due to sparse data. In contrast, for high-input datasets, where the knee plot often fails to yield a clear inflection point, the negative binomial model performs better, as the larger data volume supports more reliable parameter estimation and robust model fitting.

**Simulation Method**

To simulate UMI sequencing with varied read types and library sizes, we extended the UMIc-seq simulation framework, [**UMIC-seq/figures/SuppFig1/simulate_UMI-length-errorrate.py at master · fhlab/UMIC-seq**](https://github.com/fhlab/UMIC-seq/blob/master/figures/SuppFig1/simulate_UMI-length-errorrate.py) with key modifications. Instead of a normal distribution, we adopted a negative binomial distribution parameterized by user-defined expected count x and variance s^2^. Using the method of moments, parameters were computed as:

$r =\frac{x^{2}}{s^{2}-x}$ *,* $p=\frac{x}{s^{2}}$

Progeny UMI counts were then sampled using numpy.random.negative_binomial (Harris et al., 2020). To simulate sequencing errors, each base in the founder UMI had a chance to mutate if a randomly drawn float from [0, 1] was below the specified error rate. Possible errors included single-base insertions (A/C/G/T), deletions, and substitutions (to one of the three alternative bases). To reflect platform-specific error profiles (e.g., Illumina vs ONT), we defined configurable ratios of insertion, deletion, and substitution. A mutation pool was constructed by repeating each error type according to its specified frequency, and mutations were sampled from this pool. Counts of each error type were tracked and included in benchmarking metrics.

**Tool Parameters for Simulated Data**

**UMI-nea** requires the input file and the maximum UMI length. The tool automatically determines the edit distance threshold from the error rate and UMI length.

UMI-nea -i input -o output -l max_UMI_length -t 48 -e error_rate

**UMI-tools,** [**https://github.com/CGATOxford/UMI-tools**](https://github.com/CGATOxford/UMI-tools)**,** uses Hamming distance by default for UMI clustering. To enable indel handling, we implemented Levenshtein distance calculations by modifying the network.py module to replace the default Hamming distance with Levenshtein distance, using the *editdistance* Python package (Harris et al., 2020).

import editdistance

for umi1, umi2 in iter_umi_pairs:

if editdistance.eval(umi1, umi2) <= threshold:

To remove alignment effects, the same read alignment is used for all simulated and simulated UMI sequence attached to each read name. umi-tools group is used with default method directional and same edit distance as UMI-nea to obtain a flat file describing the read groups. The cluster labels are obtained from the unique id for the group in the output flat file.

umi_tools group -I input.bam --edit-distance-threshold=UMI-nea_edit_distance --group-out=output

**UMIc-seq,** [**https://github.com/fhlab/UMIC-seq**](https://github.com/fhlab/UMIC-seq)**,** clustering includes two steps: clustertest to identify optimal alignment thresholds based on a subset of UMIs, and clusterfull to group all UMIs with selected threshold. For UMI length < 20 bp, clustertest runs with threshold steps 5–25; for UMI length ≥20 bp: 20–40. For each threshold, clustertest reports cluster size and similarity. We modify UMIC-seq.py to record cluster threshold and similarity score to .clustertest file.

def threshold_approx(umis, ssize, left, right, step, outname):

outf = open(outname + ".clustertest", "w")

for thresh in threshholds:

outf.write(f"Threshold {thresh}: Average similarity {average_sim:.2f} and median size {median_size:.0f}\n")

outf.close()

We adopt the same criteria that UMIc-seq used for automatically selecting optimal threshold: 1) similarity score increment is less than 0.02 × UMI length, 2) threshold is greater than 0.5* UMI length. This approach can fail in some replicates when UMI length is less than 20, which will cause the threshold to be small and the number of clusters diverged from the expected. The full clustering uses the selected threshold and minimal cluster size 1 to allow singletons.

python UMIC-seq.py -T 48 clustertest -i input.fa -o output.clustertest --steps s1 s2 1

python UMIC-seq.py -T 48 clusterfull -i input.fa -o output --reads input.fastq --aln_thresh threshold --size_thresh 1 --stop_thresh 1

**Calib,** [**https://github.com/vpc-ccg/calib**](https://github.com/vpc-ccg/calib)**,** requires fastq file and UMI length. Simulated UMI sequences are prepended to the start of the same reads sequence. Calib runs with default parameters and simulated UMI length. Calib adjusts its parameters according to the read length and UMI length.

calib -f input.R1.fastq -r input.R2.fastq -l1 UMI_length -l2 0 -o output -c 48

**V-measure Calculation for Simulated Data**

V-measure scores were used to assess UMI deduplication accuracy. This entropy-based metric balances homogeneity (the uniformity of UMIs grouped from the same original molecule) and completeness (the extent to which all UMIs from a given molecule are captured within a single cluster). A score of 1 indicates perfect agreement with the ground truth, while lower scores reflect over- or under-clustering. V-measure provides a normalized and objective framework for comparing tools across varied simulated experimental conditions. Scores were computed using the *v_measure_score, homogeneity_score* and *completeness_score* functions from scikit-learn (Pedregosa et al., 2011).

**UMI Extraction and Clustering for Real TCR Sequencing Data**

UMIs were extracted using an in-house pipeline based on TCR read structure. Clustering was performed separately for each sub-gene using the four tools.

**UMI-nea** used default error rate (0.001), maximum UMI length. Edit distance is 1:

UMI-nea -i input -o output -l max_UMI_length -t 48

**umi-tools** uses the same alignments and edit distance from UMI-nea:

umi_tools group -I input.bam --edit-distance-threshold=1 --group-out=output

**UMIc-seq** followed the same threshold selection strategy as for simulations. To ensure timely completion, we applied the Miseq-derived threshold minus one to all Nextseq datasets:

python UMIC-seq.py -T 48 clustertest -i input.fa -o output.clustertest --steps 5 25 1

python UMIC-seq.py -T 48 clusterfull -i input.fa -o output --reads input.fastq --aln_thresh threshold --size_thresh 1 --stop_thresh 1

Calib uses default parameters and UMI-prepended input reads.

calib -f input.R1.fastq -r input.R2.fastq -l1 18 -l2 0 -o output -c 48

UMI-nea applied an adaptive threshold to discard low-confidence UMI clusters, whereas the other tools removed only singleton UMIs to reduce noise, as commonly recommended in UMI-based workflows (Marx, 2017).

**Post-Clustering Processing for Real TCR Sequencing Data**

Consensus reads for each UMI cluster are generated using the in-house tool (unpublished). Imseq 1.1.0, [Release IMSEQ 1.1.0 · lkuchenb/imseq](https://github.com/lkuchenb/imseq/releases/tag/v1.1.0) annotates the clonotypes from the consensus reads.

imseq -ref ref.fa -mcq 0 -mrl 75 -ma -ev 0.3 -vcl 20 -mq 0 -j 64 -o detail.txt -rlog rejects.txt -on imseq.count consensus_reads.fastq

**MiXCR Analyses** **on Real TCR Sequencing Data**

MiXCR was used under a license and obtained separately from the software authors. Analyses were performed using a built-in preset for QIAseq Human TCR Panel Immune Repertoire RNA Library Kit.

mixcr analyze qiagen-human-rna-tcr-umi-targeted-qiaseq R1.fastq R2.fastq out

**Supplementary Table 2** Run time Comparison with Multi-threading for Calib, UMI-nea, and UMIc-seq on simulated Short-read (12 bp UMI, 0.5% error rate) and Long-read (25 bp UMI, 1% error rate) data with 10,000 Founders and 100 Progeny in triplicate.

^a^Benchmarked runtimes for all tools include input file reading, UMI clustering, and output writing. Input formats vary across tools: Calib and UMIc-seq process FASTQ files and UMI-nea uses a preprocessed TSV file containing unique UMI sequences and their counts. Notably, only Calib's runtime includes the UMI extraction step, whereas UMIc-seq, and UMI-nea exclude this step from their benchmarking.

**Supplementary Table 3** V-measure and the Number of Resulting Clusters of UMI-nea Across Different UMI Lengths on Simulated Short-read (0.5% error rate) and Long-read (3% error rate) Data with 10,000 Founders and 100 Progeny.

**
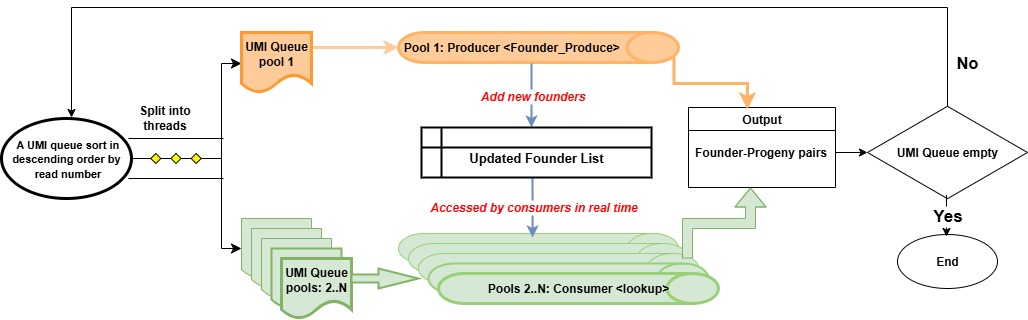
**

**Supplementary Figure 1** Workflow of UMI-nea. Unique Molecular Identifiers (UMIs) are divided into *N* pools, corresponding to *N* computational threads. A single producer thread and multiple consumer threads operate in synchronization to update founder–progeny pairs, with only the producer thread responsible for updating the founder list and identifying the closest founder of each progeny UMI.

(A) Calib


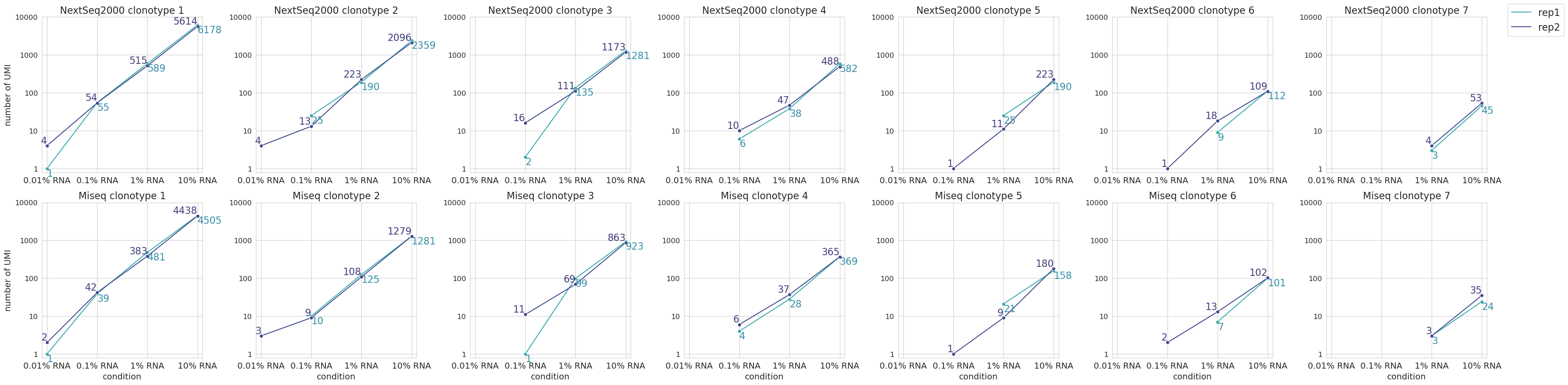


(B) UMIc-seq


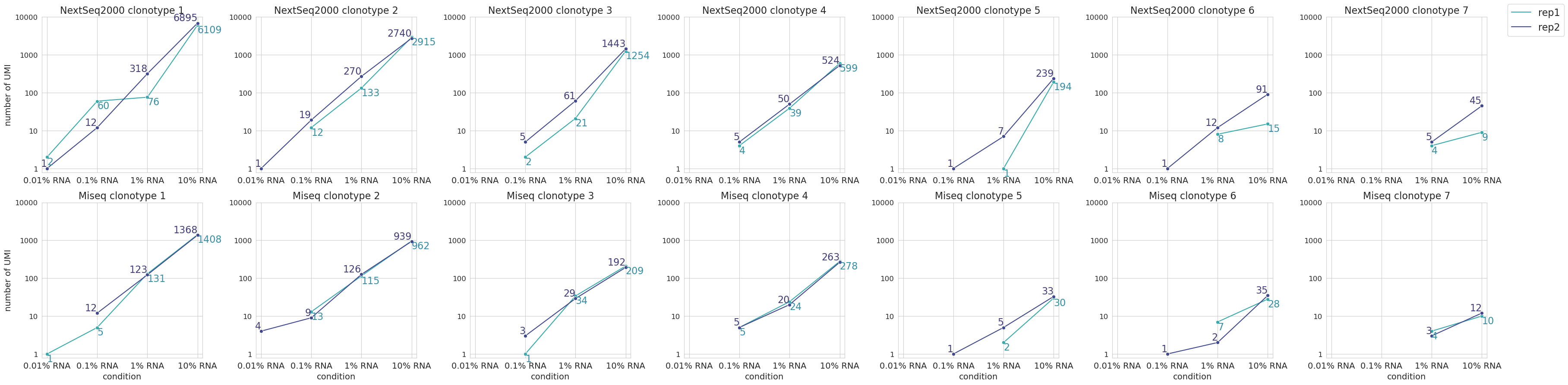


(C) UMI-tools


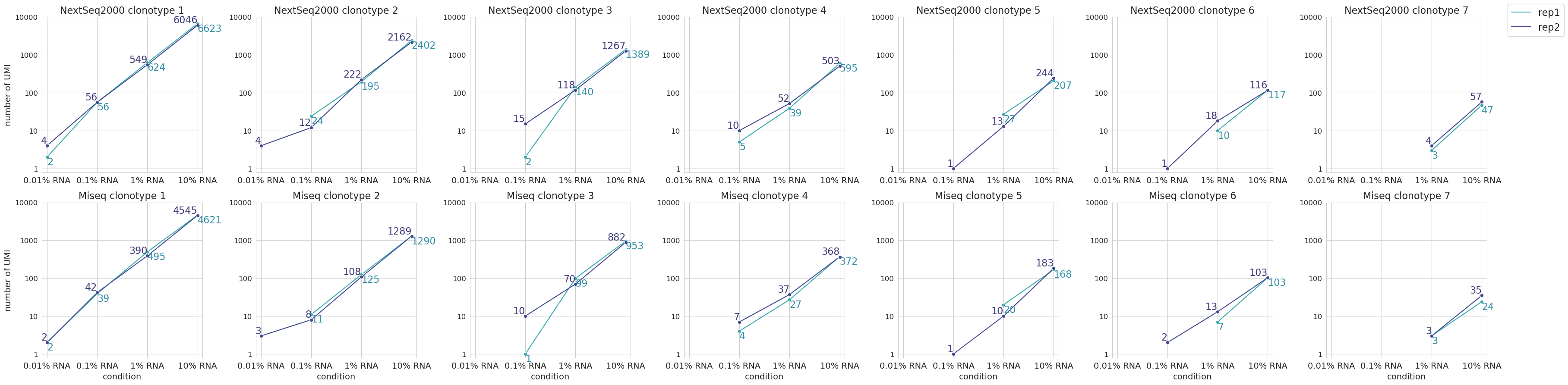


(D) UMI-nea


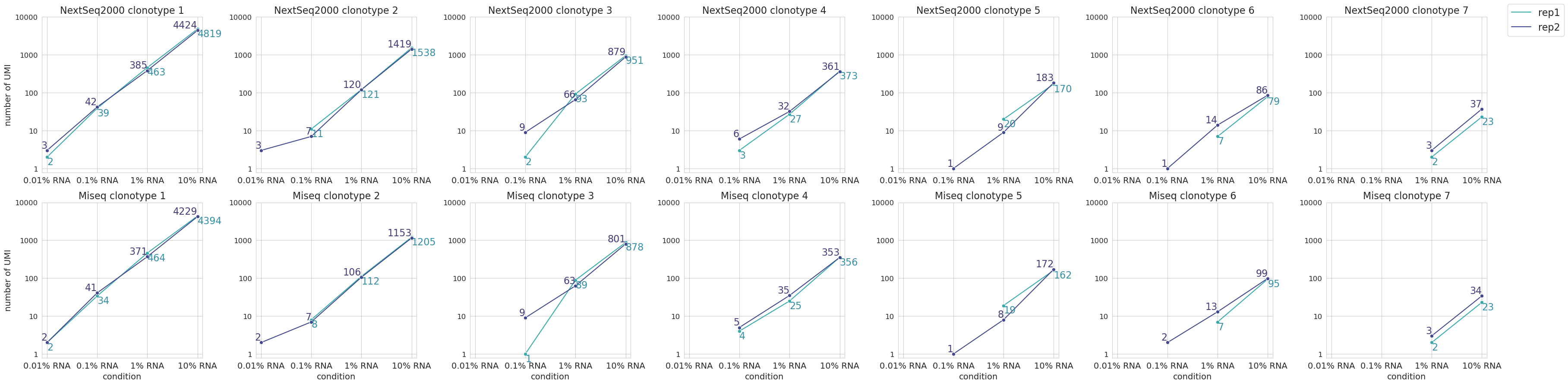


(E) MiXCR


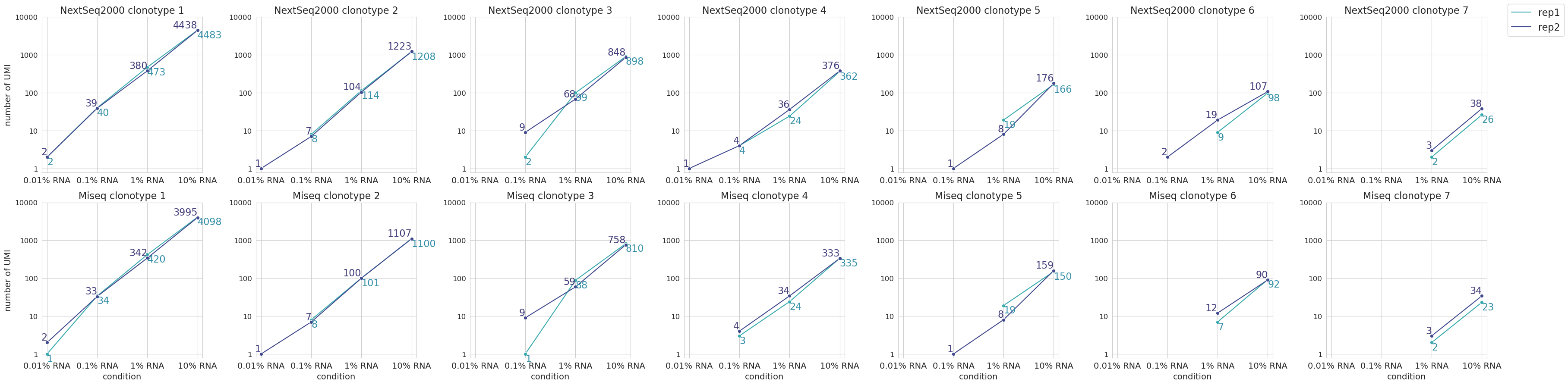


**Supplementary Figure 2** Quantification of 7 Clonotypes in Real TCR Sequencing Data with UMI-nea, umi-tools, UMIc-seq, Calib and MiXCR on Nextseq2000 and Miseq platform. (A) Calib (B) UMIc-seq (C) UMI-tools (D) UMI-nea (E) MiXCR.

clonotype 1: TRB:TRBV5-6:TGTGCCAGCAGCCGAGGGGAACCCCCCTGGACCTACAATGAGCAGTTCTTC:TRBJ2-1

clonotype 2: TRD:TRAV23/DV6:TGTGCAGCAAGCAAAGCTGCCGGTTTGACAGCACAACTCTTCTTT:TRDJ2

clonotype 3: TRB:TRBV12-3:TGTGCCAGCAGTTTCTCGACCTGTTCGGCTAACTATGGCTACACCTTC:TRBJ1-2

clonotype 4: TRG:TRGV9:TGTGCCTTGTGGGAGCCCCCCCCCTCGAATTATTATAAGAAACTCTTT:TRGJ2

clonotype 5: TRB:TRBV2:TGTGCCAGCAAGACTACCCAAAGCACAGATACGCAGTATTTT:TRBJ2-3

clonotype 6: TRA:TRAV8-4:TGTGCTGTGAGTGATCTCGAACCGAACAGCAGTGCTTCCAAGATAATCTTT:TRAJ3

clonotype 7: TRA:TRAV13-2:TGTGCAGAGAATATCGGAACTGGGGCAAACAACCTCTTCTTT:TRAJ36

Cell line 1 contains clonotype 1, 2 and 4; Cell line 2 contains clonotype 3 and 6; Cell line 3 contains clonotype 5 and 7.

**Supplementary Figure 3** Coefficient of variation (CV) of Estimated TCR Molecule Counts Across Four Technical Replicates. CVs are shown for Calib, UMIc-seq, UMI-tools, UMI-nea and MiXCR, highlighting variability in quantification reproducibility. UMI-nea and MiXCR exhibited the lowest CVs, indicating more consistent performance across replicates.


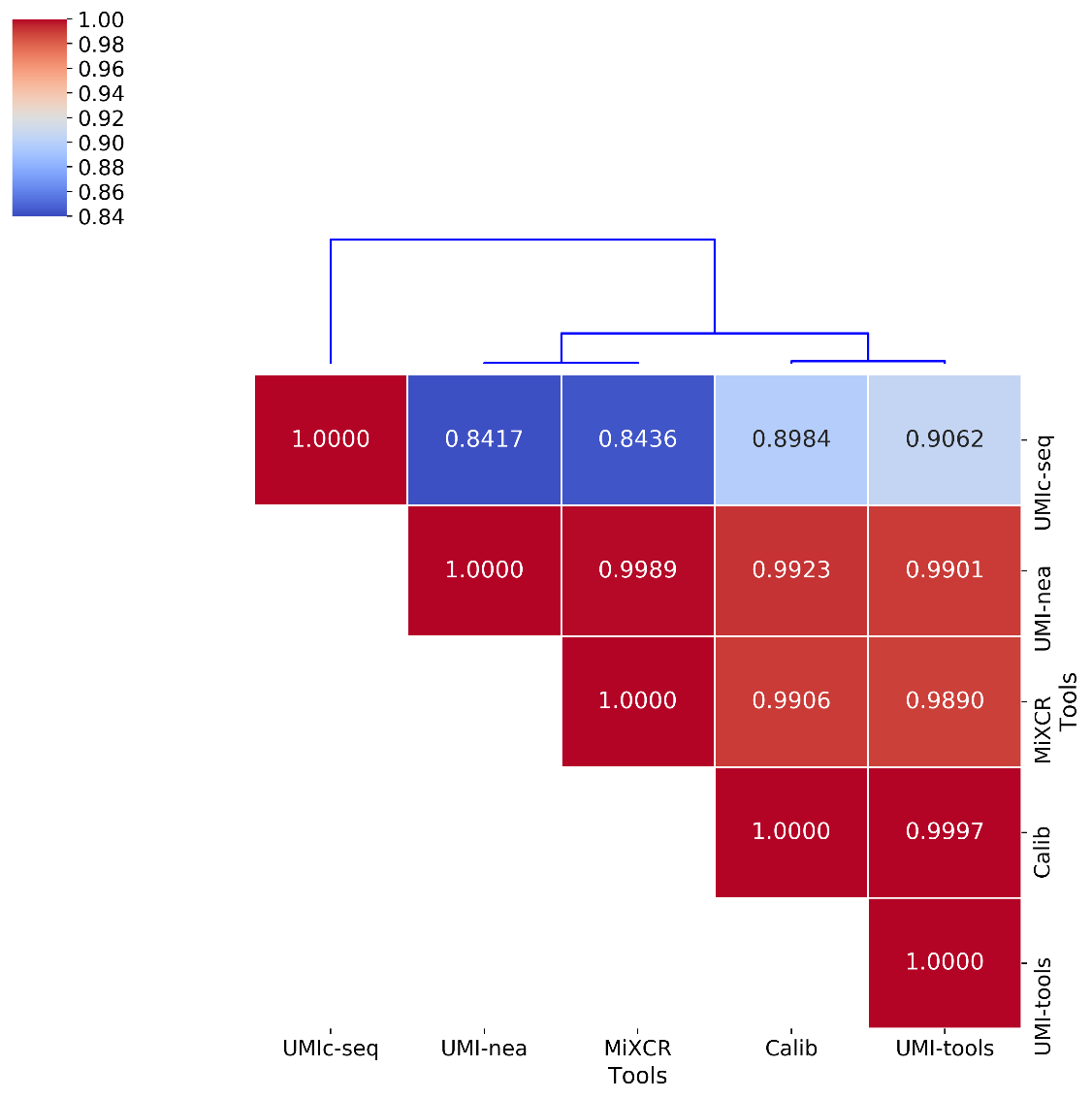


**Supplementary Figure 4**  Hierarchical clustering and Pearson correlation heatmap of TCR Molecule Count Estimates Across Different Tools. Pearson correlation was calculated on the molecule counts of seven TCR clonotypes across various experimental conditions and five tested tools to identify clustering patterns among tools producing similar estimates under each condition.

**Supplementary References**

Han RH. editdistance: fast implementation of edit distance (Levenshtein distance). GitHub; 2018. Available from: <https://github.com/roy-ht/editdistance>

Harris CR, Millman KJ, van der Walt SJ, Gommers R, Virtanen P, Cournapeau D, et al. Array programming with NumPy. Nature. 2020;585(7825):357–362.

Pedregosa F, Varoquaux G, Gramfort A, Michel V, Thirion B, Grisel O, Blondel M, et al. Scikit-learn: machine learning in Python. J Mach Learn Res. 2011;12:2825–2830.

Waskom, M. L., (2021). seaborn: statistical data visualization. Journal of Open Source Software, 6(60), 3021, https://doi.org/10.21105/joss.03021.

Zheng GXY, Terry JM, Belgrader P, et al. Massively parallel digital transcriptional profiling of single cells. Nat Commun. 2017;8:14049. <https://doi.org/10.1038/ncomms14049>
